# Control of Earth-like magnetic fields on the transformation of ferrihydrite to hematite and goethite

**DOI:** 10.1038/srep30395

**Published:** 2016-07-26

**Authors:** Zhaoxia Jiang, Qingsong Liu, Mark J. Dekkers, Vidal Barrón, José Torrent, Andrew P. Roberts

**Affiliations:** 1 State Key Laboratory of Lithospheric Evolution, Institute of Geology and Geophysics, Chinese Academy of Sciences, Beijing 100029, P.R. China; 2 Laboratory for Marine Geology, Qingdao National Oceanography Laboratory for Marine Science and Technology, Qingdao, P.R. China; 3 Department of Earth Sciences, Paleomagnetic Laboratory ‘Fort Hoofddijk’, Faculty of Geosciences, Utrecht University, Budapestlaan 17, 3584 CD Utrecht, The Netherlands; 4 Departamento de Agronomía, Universidad de Córdoba, Edificio C4, Campus de Rabanales, 14071 Córdoba, Spain; 5 Research School of Earth Sciences, Australian National University, Canberra, ACT 2601, Australia

## Abstract

Hematite and goethite are the two most abundant iron oxides in natural environments. Their formation is controlled by multiple environmental factors; therefore, their relative concentration has been used widely to indicate climatic variations. In this study, we aimed to test whether hematite and goethite growth is influenced by ambient magnetic fields of Earth-like values. Ferrihydrite was aged at 95 °C in magnetic fields ranging from ~0 to ~100 μT. Our results indicate a large influence of the applied magnetic field on hematite and goethite growth from ferrihydrite. The synthesized products are a mixture of hematite and goethite for field intensities <~60 μT. Higher fields favour hematite formation by accelerating ferrimagnetic ferrihydrite aggregation. Additionally, hematite particles growing in a controlled magnetic field of ~100 μT appear to be arranged in chains, which may be reduced to magnetite keeping its original configuration, therefore, the presence of magnetic particles in chains in natural sediments cannot be used as an exclusive indicator of biogenic magnetite. Hematite vs. goethite formation in our experiments is influenced by field intensity values within the range of geomagnetic field variability. Thus, geomagnetic field intensity could be a source of variation when using iron (oxyhydr-)oxide concentrations in environmental magnetism.

Hematite and goethite are the two most abundant iron oxides in soils and sediments from many climatic zones; they are, however, generated in competition with each other^1^,^2^,^3^. Therefore, the ratio of these two minerals is often used to indicate climatic and environmental variation in soil records^4^,^5^ and in marine sediments^6^,^7^. Hematite and/or goethite growth from precursor ferrihydrite is controlled by many factors, such as temperature, pH, humidity, oxygen fugacity, and the presence of other ions in the system (e.g., Al and Si)^8^,^9^,^10^,^11^,^12^,^13^. Generally, warm and dry conditions favour hematite formation^14^,^15^, while more humid conditions favour goethite formation^16^,^17^. Neutral pH and temperatures >~90 °C induce hematite formation while acid or alkaline solutions at temperatures <~80 °C yield goethite^17^. Increasing temperature and Al content favours hematite formation while an alkaline solution with high pH favours goethite formation^11^. The presence of dissolved phosphate and/or silicate may retard hematite and goethite formation^8^,^13^. In addition, hematite crystallizes along the direction of an applied magnetic field and faithfully records that field direction^18^,^19^. Therefore, it is reasonable to ask whether growth of hematite and goethite is influenced by the ambient geomagnetic field, which undergoes considerable variation both spatially and temporally^20^,^21^. However, it is presently unknown whether magnetic fields with different, Earth-like, intensities exert control on magnetic mineral growth. The range of field intensities up to ~100 μT is relevant in this context.

Possible magnetic field control has been studied in relation to formation of magnetite^22^,^23^ and greigite (Fe_3_S_4_)^24^. The saturation magnetization (*M*_s_), saturation remanent magnetization (*M*_r_), and coercive force (*B*_c_) are markedly higher for samples synthesized in strong applied magnetic fields (0.25 T in the cited experiments) than for samples synthesized in the much weaker geomagnetic field^23^. It was also found that chain-like magnetic particles can grow under the influence of an external magnetic field^23^. For magnetite, higher magnetic fields (~0.35 T) tend to result in formation of larger and more crystalline particles^23^. *He et al.*^24^ investigated the magnetic-field-induced phase-selective formation of ferrosulphide microrods for materials applications. This was done using a hydrothermal process, where ammonium iron sulphate hexahydrate and different sulphur precursors were dissolved in distilled water and stirred vigorously for 30 min, and then aged in the presence or absence of a magnetic field (45 mT) at 180 °C in a Teflon vessel with circular disk-shaped magnets at the top and bottom of a stainless steel autoclave. Pure greigite was produced in the 45 mT field, whereas a mixture of Fe_3_S_4_ and FeS_2_ was produced in the much weaker geomagnetic field. Therefore, these authors proposed that introduction of a strong magnetic field to the reaction system preferentially induced formation of a pure magnetic phase over a mixture with a paramagnetic phase^24^.

The magnetic fields used in previous studies were in the millitesla (mT) range, which is larger than typical geomagnetic field intensities. Also, these studies focused on strongly magnetic minerals with materials applications in mind. Thus, the cited results cannot be extrapolated directly to natural soil and sediment samples. In this study, we synthesized a series of hematite and goethite samples under a range of controlled Earth-like fields to investigate a possible field dependence of hematite and goethite formation.

## Results

### Structure and morphological properties

**Figure 1:**
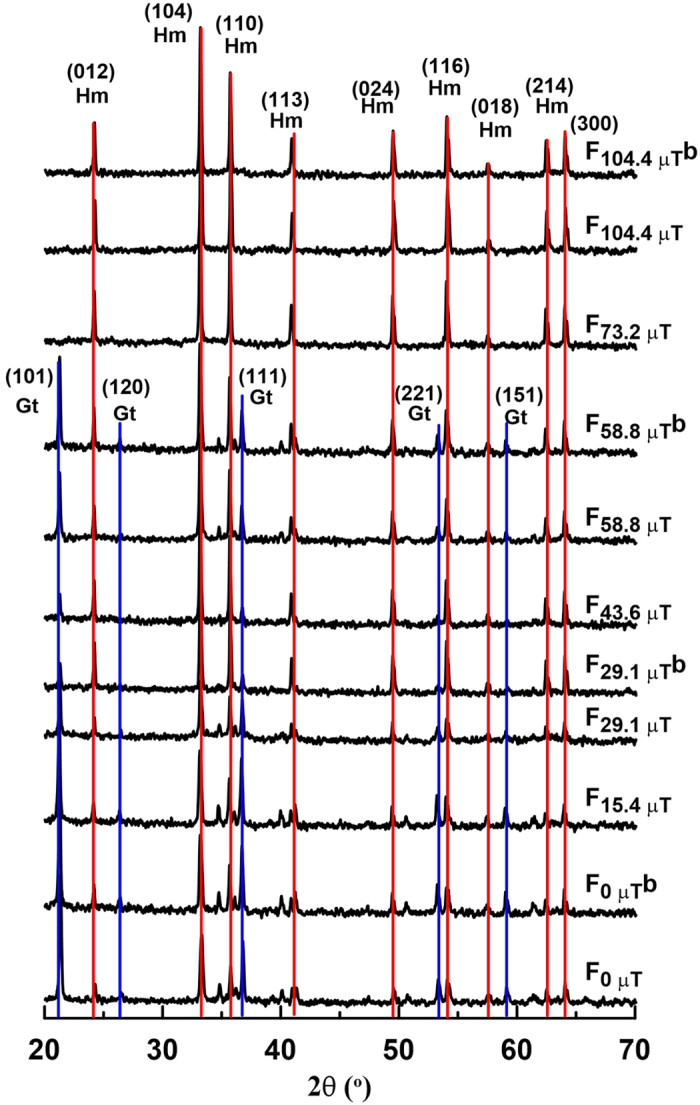
X-ray diffractograms for samples synthesized in different applied magnetic fields. The red and blue lines represent the characteristic lattice reflections for hematite and goethite, respectively, where Hm and Gt stand for hematite and goethite respectively, and numbers between the brackets are Miller indices for respective reflections for goethite and hematite. Numbers in the sample labels represent the field intensity, e.g., F_15.4 μT_ represents a sample synthesized in a 15.4 μT field.

**Figure 2:**
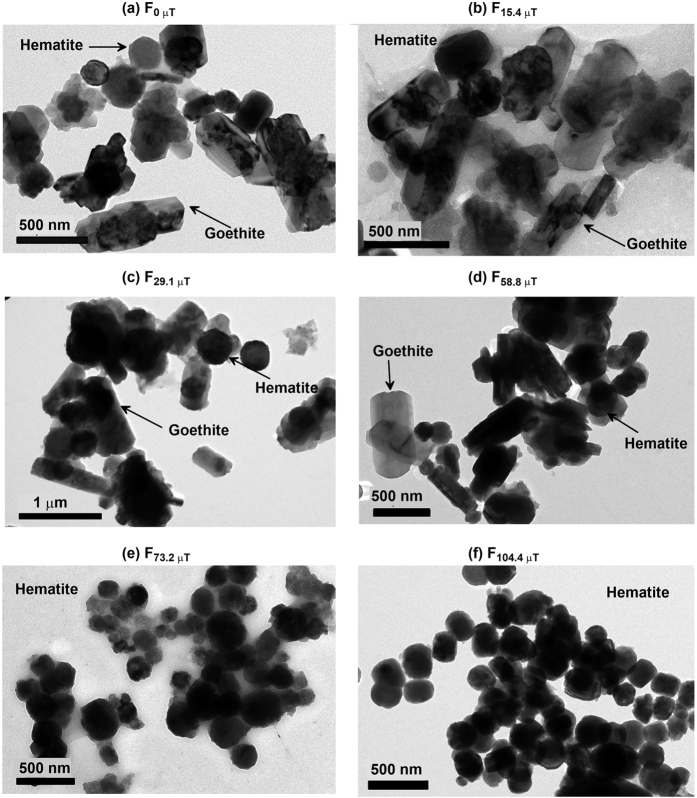
Transmission electron microscope (TEM) images of samples grown in different magnetic fields. Sample labelling is as in Fig. 1.

From X-ray diffractograms (Fig. 1), it is clear that the magnetic field strongly affects the transformation of ferrihydrite to hematite and goethite. For fields below ~60 μT, hematite and goethite coexist. In contrast, above 60 μT, hematite is dominant and goethite is undetectable. Based on XRD results, we infer that the rod-shaped particles in TEM observations (Fig. 2) are goethite, while the relatively small oblate grains are hematite. For growth fields <~60 μT, goethite is observed commonly with an axial length of up to 700 nm and width of up to 200 nm (Fig. 2a–d). Hematite grains with a particle size of ~300 nm are dispersed around goethite. When the applied field during synthesis exceeds ~100 μT, most hematite particles appear to be arranged in chains (Fig. 2f). The size of individual platelets is remarkably uniform at ~260 nm.

### Magnetic Properties

**Figure 3:**
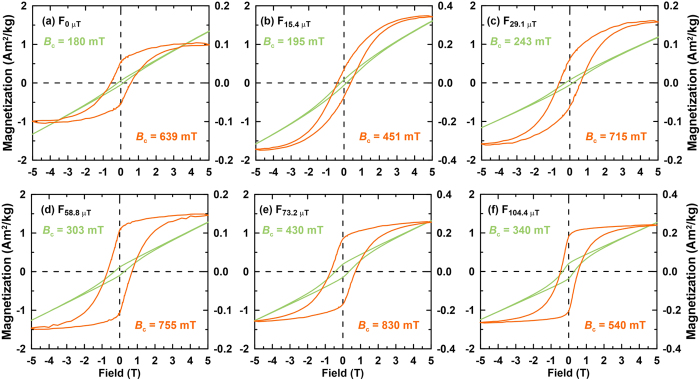
Hysteresis loops measured at room temperature before (green) and after high-field slope correction (orange) for samples grown in different magnetic fields. *B*_c_ values are indicated in green and orange, respectively. The left-hand ordinate axis refers to hysteresis loops before slope correction while the right-hand axis represents those after slope correction. Sample labelling is as in Fig. 1.

**Figure 4:**
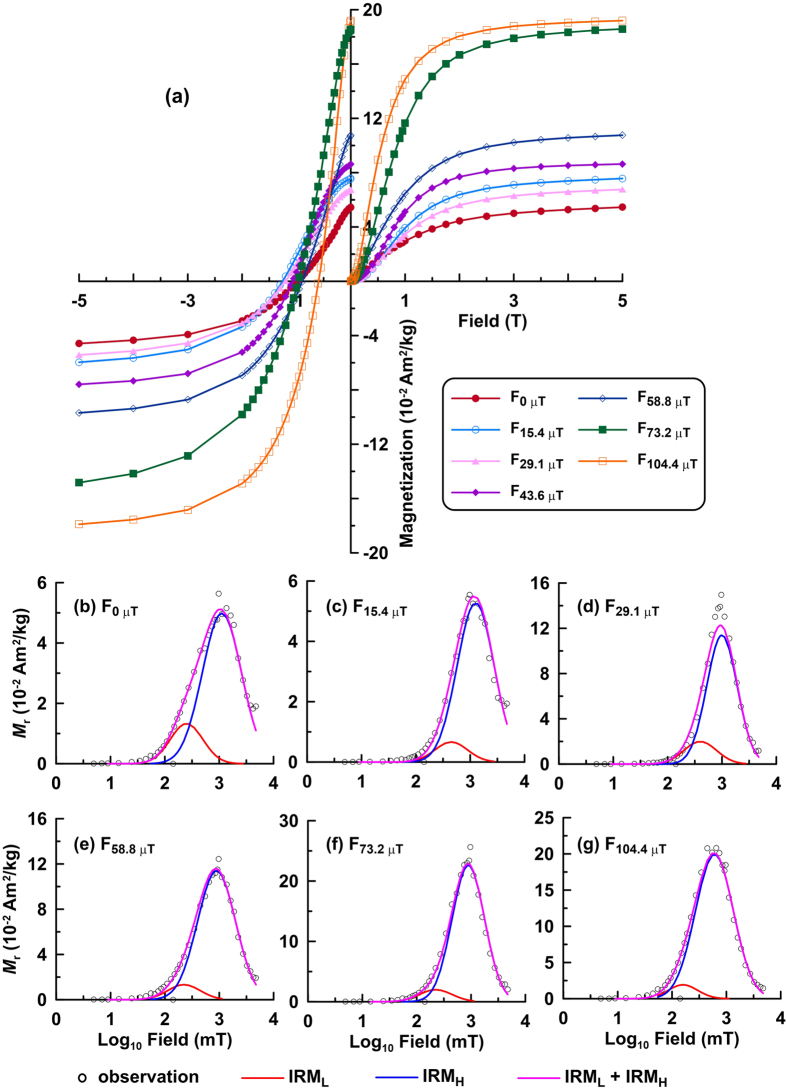
(**a**) IRM acquisition curves and DC demagnetization curves for samples grown in different applied fields. (**b–g**) IRM component analysis for hematite powder samples, where the red, blue, and pink lines indicate, respectively, the low coercivity component (IRM_L_), high coercivity component (IRM_H_), and the sum of the two components (IRM_L_ + IRM_H_). Open circles represent the measured data.

The samples are not saturated magnetically even in a 5 T field (Figs 3 and 4a). The hysteresis loops before slope correction also become wider with increasing applied field during synthesis, and *B*_c_ and *M*_r_ values for the samples generally increase with increasing applied field during synthesis (Fig. 3). IRM_@5T_ increases gradually from 6 × 10^−2^ to 19 × 10^−2 ^Am^2^/kg with increasing applied field during synthesis. IRM_@5T_ for samples that grew in fields <~60 μT is smaller than the SIRM of typical hematite (~0.1 Am^2^kg^−1^), which may be attributed to goethite contamination and lower overall particle crystallinity, or to different synthesis conditions. IRM component analyses indicate the presence of two magnetic components, a relatively low coercivity (L) component (IRM_L_) with a mean coercivity of around 100–300 mT, and a high coercivity (H) component (IRM_H_) with a mean coercivity of around 1 T (Fig. 4b–g, Table 1). Pure goethite (i.e., that lacks cation substitution) has large coercivities and cannot be saturated even in a 57 T field^25^. Therefore, the two identified coercivity components are attributed to hematites with different coercivities. Apparently, the IRM of these samples is dominated by IRM_H_, which contributes 82% to 95% of the IRM (Table 1). IRM_L_ may be attributed to fine particles that are also produced through the aging in solution. The IRM of these quasi-superparamagnetic particles is sensitive to the process of thermal activation which leads to a softer IRM component, IRM_L_, next to the dominant IRM_H_^26^.

**Figure 5:**
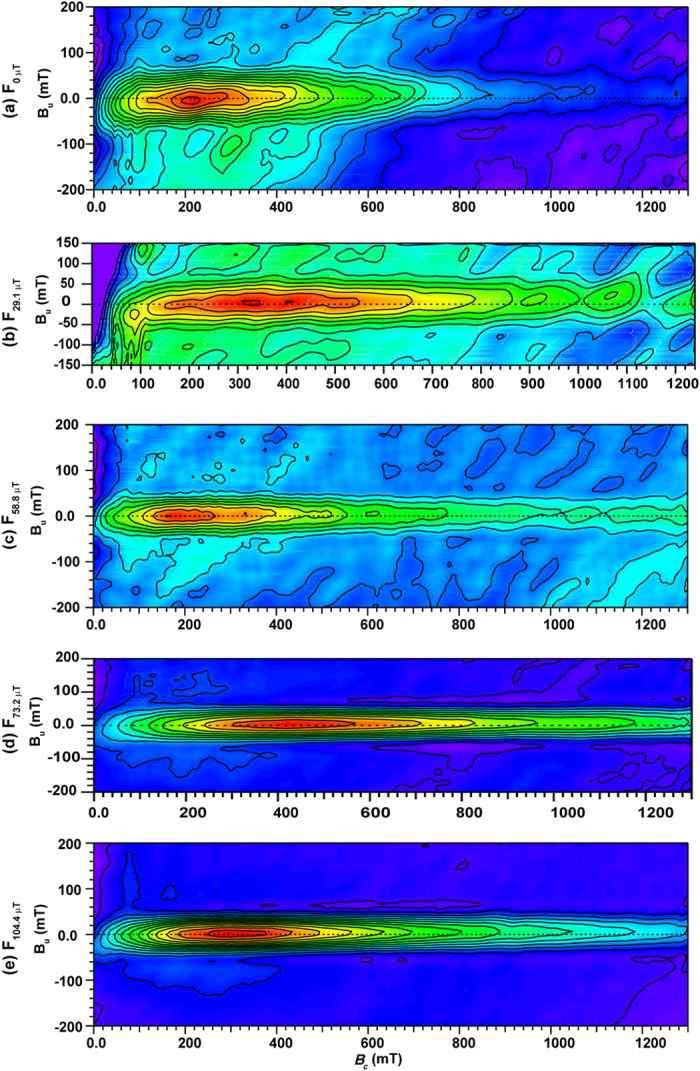
FORC diagrams for sample powders grown in different magnetic fields. The data were calculated with the FORCinel v1.18 software^60^ and were processed with a smoothing factor of 7. Coloring indicates the relative FORC contour density with blue zero density and red maximal density. *B*_u_ is the interaction field, *B*_c_ the coercivity field; for sample labels cf. Fig. 1.

FORC distributions for all of the synthesized samples are elongated with closed contours, which are indicative of stable single domain (SD) particles (Fig. 5)^27^,^28^. For samples F_0 μT_ and F_29.1 μT_, the interaction field is ~50 mT, which is determined from the full width of the FORC distribution at half of its peak value. However, as the applied field during synthesis increases, the vertical spread of the FORC distribution becomes narrower and stabilizes at ~40 mT. Goethite is not detected in the FORC diagrams because of its large coercivity (>1 T). Significant magnetostatic interactions tend not to be important in natural and some synthetic goethite and hematite samples because the weak spontaneous magnetization of these minerals makes them less susceptible to strong interactions^29^. The interaction field distributions evident in Fig. 5 are, therefore, a likely consequence of the close packing of magnetic particles in our measurements.

**Figure 6:**
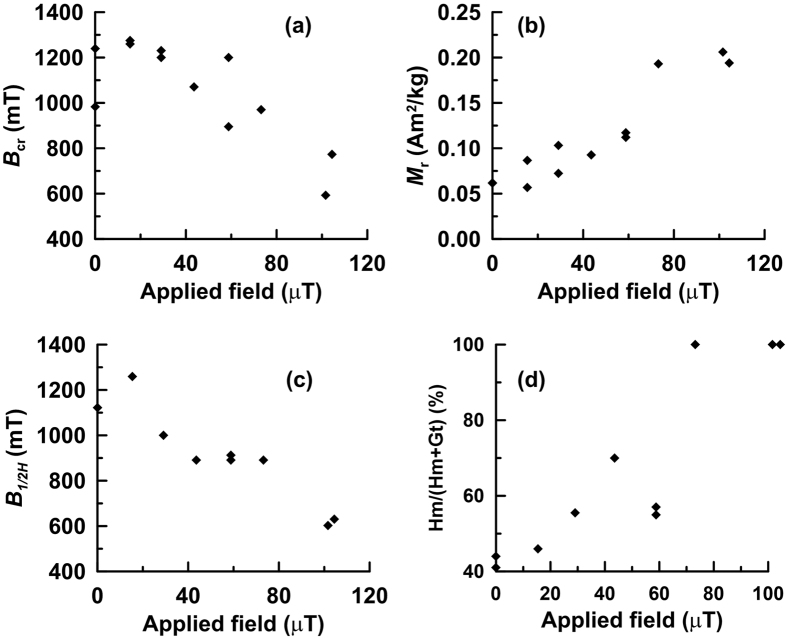
Some characteristic parameters plotted versus the applied magnetic field in which samples were grown. (**a**) Coercivity of remanence (*B*_cr_) vs. applied field; (**b**) saturation remanent magnetization (*M*_r_) vs. applied field; (**c**) *B*_1/2_ of IRM_H_ (*B*_1/2H_) vs. applied field; (**d**) Hm/(Hm + Gt), calculated from XRD data, vs. applied field. In each subfigure, the applied field is the magnetic field in which each synthesis was performed.

Hysteresis parameters for our samples are summarized in Fig. 6. *B*_cr_ decreases with increasing applied field during synthesis (Fig. 6a). In contrast, *M*_r_ increases with field in a quasi-linear trend (Fig. 6b). The median coercivity (*B*_1/2_) of IRM_H_ is denoted by *B*_1/2H_, which decreases linearly with increasing field applied during synthesis in accord with *B*_cr_ (Fig. 6c). This further indicates that IRM_H_ is the dominant component at higher applied fields during synthesis. Hm/(Hm + Gt) calculated from XRD data increases with applied field during synthesis up to ~60 μT, and reaches ~100% when applied fields during synthesis are >~60 μT (Fig. 6d). This demonstrates that the hematite content increases with increasing applied field during synthesis.

## Discussion

### Control mechanism of Earth-like fields on hematite/goethite formation

A fair number of synthesis pathways have been proposed for hematite and goethite formation, such as hydrolysis of acid solutions of Fe(III) salts, transformation of ferrihydrite, oxidative hydrolysis of Fe(II) salts, the gel-sol method, and several others^8^,^9^,^10^,^11^,^12^,^30^. Formation reactions of goethite and hematite from precursor ferrihydrite suspension are different. Goethite forms by precipitation from solution after ferrihydrite dissolution, while hematite formation is preceded by ferrihydrite aggregation and hematite nucleation within these aggregates as a solid phase reaction^8^,^10^. However, it is proposed recently that goethite rods can be formed from ferrihydrite through phase transformation followed by oriented aggregation^31^,^32^, where ferrihydrite suspension has been dialyzed against Milli-Q water to remove the solvent and/or surface molecules, which favours an irreversibly oriented aggregate formation^32^. However, in natural environments, it is difficult to remove the solvent. Given that ferrihydrite suspension to hematite or goethite pathway is so prominent in natural environments^1^,^2^, we, therefore, primarily focus on the influence of magnetic fields on the final product synthesized via this route.

In general, higher or lower pH and lower temperature (<80 °C) favour goethite formation while neutral pH and higher temperature (>90 °C) favour hematite formation since dissolution of ferrihydrite is retarded at neutral pH^10^,^17^. In our experiments, NaHCO_3_ was added to bring pH to ~7 at room temperature and aging temperature was fixed at 95 °C to inhibit goethite formation. The actual pH at 95 °C is not easy to measure, but it will be the same for all samples. However, substantial goethite still formed at 95 °C, where its concentration decreased with increasing applied field during synthesis (Figs 1,2 and 6). This demonstrates that the applied field strength during synthesis plays a significant role in the formation of these two minerals, even in weak Earth-like fields.

**Figure 7:**
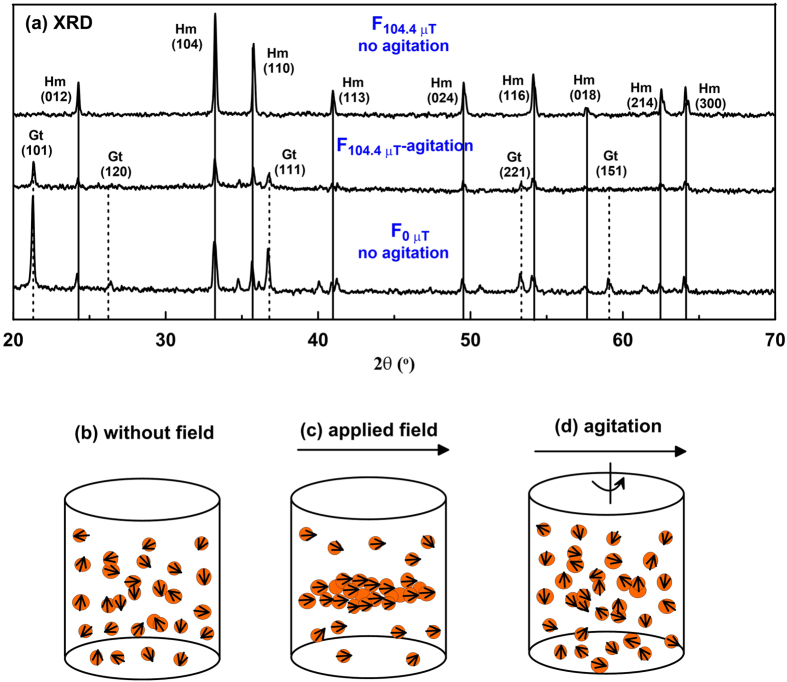
(**a**) X-Ray diffractograms of samples grown in a ~0 μT field, and a 104.4 μT field with and without agitation; (**b–d**) the model proposed for ferrihydrite aging (**b**) without an applied field, (**c**) in an applied field and (**d**) with agitation in an applied field, where the orange circles with arrows represent ferrimagnetic ferrihydrite particles, and the arrows above the cylinders indicate the applied field direction.

Based on our results and the different formation mechanisms of hematite and goethite, we infer that an applied magnetic field should favour ferrihydrite aggregation. To test this inference, we synthesized a sample under the same conditions as sample F_104.4 μT_, but stirred the suspension for 5 minutes at 5-hour intervals with a glass stick to disturb ferrihydrite particle aggregation. This sample is labeled ‘F_104.4 μT_-agitation’. XRD results indicate that the final product is a mixture of hematite and goethite (Fig. 7a). This means that, although aging took place in a magnetic field of ~100 μT, the preference for hematite formation was disturbed by the agitation. Therefore, we propose a model in which ferrihydrite can aggregate or dissolve depending on the solution conditions (e.g., pH, temperature, etc.). Without an applied field (Fig. 7b), hematite and goethite form simultaneously (apparently the two formation mechanisms compete). However, if a magnetic field is applied above a certain threshold value, ferrimagnetic ferrihydrite, which is an intermediate transformation product in the reaction from ferrihydrite to hematite^33^, can aggregate parallel to the field direction (Fig. 7c) and hematite will form preferentially. Even in a relatively high applied field, this aggregation can be disrupted by intermittent agitation (Fig. 7d); then both hematite and goethite are produced (Fig. 7a). The suggested mechanism is a preliminary hypothesis. Recently, it is proposed that goethite rods can be formed from ferrihydrite through phase transformation followed by oriented aggregation^31^,^32^, but magnetic field effect was not involved. So no other studies exist along these lines, more explicit explanations for the apparent control of an ambient magnetic field on magnetic mineral formation are needed, including further experimentation and molecular dynamics calculations^34^,^35^.

### Geological significance

Transformation of ferrihydrite to hematite or goethite in soil solutions is controlled by various environmental factors (pH, water activity, temperature, surface poisoners, etc.)^8^,^9^,^10^,^11^,^12^,^13^,^36^. For example, Torrent and Guzmán^11^ reported that hematite is favoured over goethite by decreasing water activity. Furthermore, they reported that higher ionic strength, i.e., a more concentrated solution, also influences the transformation pathway, e.g., calcium and magnesium favour hematite over goethite. Thus, hematite can form and will be favoured over goethite in natural environments with low water activity, for instance dry or saline soils or sediments^37^,^38^. In addition, mobile Al in the form of Al^3+^, Al(OH)^2+^ or Al(OH)_2_^+^, and Si in the form of H_4_SiO_4_ in the solution may also promote hematite formation by reducing ferrihydrite solubility, which in turn favours the internal hematite crystallization mechanism over the via-solution mechanism required for goethite crystallization^2^,^39^. However, it is evident from our results that hematite and goethite concentration is controlled not only by environmental factors but also by applied field strength. *M*_r_ increases with applied field strength during synthesis (Figs 3,4a and 6b,c), which may be caused by varying hematite and goethite contents that formed in competition. In addition, the relative hematite content (Hm/(Hm + Gt)) increases with applied field strength (Fig. 6d). Thus, geomagnetic field intensity variations could play an important role in the selective formation of iron oxides, which has been largely overlooked until now.

For a dipole field, the field intensity at the poles is twice that at the equator. Typical Earth-like values are ~30 μT at the equator and ~60 μT at the poles^40^,^41^. The minimum intensity of the present-day geomagnetic field, which occurs within the South Atlantic Magnetic Anomaly region, is ~22 μT while the highest field intensity is ~64 μT in Siberia and in the Southern Ocean and Antarctica south of Australia^40^. In addition, non-transitional paleointensities over the past 300 Ma typically range from ~20 to ~80 μT^21^,^42^. Therefore, geomagnetic intensity influence on iron oxide formation could be a source of variation when using magnetic concentration proxies to investigate climate variations on long time scales or spatial scales, e.g., studies of dry versus humid variability on the Chinese Loess Plateau^4^,^5^, paleoclimatic studies of marine sediments^7^,^8^, or spatial variations of hematite and goethite contents^14^.

### Indicators for identification of biogenic magnetite

Biogenic magnetic minerals (i.e., magnetite or greigite magnetosomes) have widespread occurrence in natural environments and make significant contributions to the paleomagnetic and paleoenvironmental record of sediments, because of their stable SD remanence and sensitivity to geochemical conditions that control their growth and preservation^43^,^44^,^45^. Discrimination of these biogenic magnetic minerals is important for environmental interpretations, especially in relation to assessing records of ancient magnetosome occurrences^46^. Such magnetofossils have distinct crystal morphologies, narrow particle size distributions, and chain structures; thus, criteria have been developed to identify their presence based on these features^29^,^44^,^46^,^47^,^48^,^49^. TEM observation of aligned magnetite crystals in chains and their general cuboidal shape are interpreted to indicate a bacterial origin^44^,^47^. Magnetic techniques for magnetofossil detection are rapid and nondestructive, and include low temperature magnetism^50^,^51^, coercivity analysis of IRM acquisition curves^52^, and FORC diagrams and ferromagnetic resonance spectroscopy (FMR)^29^,^44^,^47^,^48^,^49^. Magnetosome chain structures produce FMR spectra with multiple derivative maxima, asymmetry (A) <1 and an effective g-factor (g_eff_) <2.12^44^,^47^,^48^. FORC analysis provides an excellent tool for isolating the biogenic component as a ‘central ridge’ signature with a peak in the switching field distribution between ~20 and 60 mT^29^,^44^,^49^.

Our results indicate that hematite synthesized in a magnetic field of ~100 μT can also have chain-like arrangements with unique particle size of around 260 nm. If these samples were preserved in short-lived periods with reducing conditions, they may be reduced to magnetite but inherit the same shape. For example, in the presence of iron-reducing bacteria and organic matter, hematite in well-drained soils can be transformed to magnetite through fermentation^53^,^54^ or be reduced to magnetite during burning and maintain its morphology, as discussed by Jiang *et al.*^55^. This kind of abiotic chain-like magnetite may confuse magnetofossil identification, and would lead to erroneous paleoenvironmental interpretation^56^. Thus, attention should be paid to this aspect when assessing probable records of biogenic magnetic minerals.

To summarize, our results indicate that Earth-like magnetic fields affect hematite and goethite growth. Aging of ferrihydrite at 95 °C yields as the final synthesis product a mixture of hematite and goethite for field intensities <~60 μT. The hematite content increases with increasing applied field during synthesis at the expense of the goethite content. Higher fields favour hematite formation, which is illustrated by decreased *B*_cr_ and increased *M*_r_ values. Hematite formation from ferrihydrite is a solid-phase reaction, while goethite is precipitated from solution after ferrihydrite dissolution. Magnetic fields are interpreted to accelerate ferrihydrite aggregation via a ferrimagnetic intermediate phase. This implies that the effect of geomagnetic field intensity variations on iron oxide formation could be relevant when evaluating the climatic significance of their concentration variations, e.g., in loess/paleosol sequences. Furthermore, hematite particles grown in magnetic fields of ~100 μT appear to be arranged in chains with individual particles having a unique size of around 260 nm. Chain-like magnetic particle arrangements cannot, therefore, be used as an exclusive indicator of biogenic magnetofossils.

## Methods

### Sample preparations

Fourteen samples starting from pure ferrihydrite (Fe_8.2_O_8.5_(OH)_7.4_) were prepared under similar conditions. Ferrihydrite was synthesized by mixing 100 mL of 0.5 M Fe(NO_3_)_3_ and 100 mL of 2 M NaOH at room temperature. Subsequently 50 mL of 1 M NaHCO_3_ was added to bring the pH to ~7 at room temperature. The deionized water used for the solutions was heated to 95 °C in advance to inhibit goethite formation. The ferrihydrite was aged under controlled temperature and field conditions. The temperature of the suspensions was maintained at 95 ± 3 °C in a small furnace equipped with a digital thermometer. A controlled magnetic field was obtained with two sets of cubic Helmholtz coils (1 metre × 1 metre), which were aligned with the declination of the ambient laboratory field. One pair of coils was set horizontally to produce the vertical field component, while the other pair was set vertically to produce the horizontal component. The fields were set to ~0 (which probably deviated from 0 μT but not by more than 100–200 nT), 15.4, 29.1, 43.6, 58.8, 73.2, and 104.4 μT, respectively. The samples are labeled with their growth field intensities as F_0 μT_, F_0 μT_b, F_15.4 μT_, F_15.4 μT_b, F_29.1 μT_, F_29.1 μT_b, F_43.6 μT_, F_43.6 μT_b, F_58.8 μT_, F_58.8 μT_b, F_73.2 μT_, F_73.2 μT_b, F_104.4 μT_, and F_104.4 μT_b, where sample labels ending with ‘b’ are replicate samples and ‘F’ is the abbreviation for ‘field’. In order to determine when the reaction was completed, 10 mL of suspension was extracted every five hours to measure magnetic susceptibility variations in a trial experiment as described by Jiang *et al.*^19^. After aging, samples were washed free of salts by centrifuging the suspension with deionized water until the electrical conductivity of the equilibrium solution became <0.01 dS/m (dS/m: 10^−2^ Siemens per meter). Finally, the samples were dried in an oven at 40 °C.

### XRD and TEM analysis

Sample purity was verified with powder X-ray diffraction (XRD) with a D/MAX-2400XRD instrument with monochromatic Cu*Κ*α radiation, a scan step size of 0.017° 2θ, and a scan step time of 1 s. The receiving slit size was 0.1 mm. Semi-quantitative values for hematite and goethite proportions within samples were calculated from XRD data based on a standard curve for hematite and goethite mixtures, which was calibrated from the area underneath the diffraction peaks from several mixtures with known hematite and goethite contents^57^. Transmission electron microscope (TEM) images were obtained with a JEM-2010 microscope operating at 100 kV to examine particle morphology and possible grain size variation as a function of applied field during sample synthesis.

### Magnetic measurements

Both acquisition curves of the isothermal remanent magnetization (IRM) and back-field curves with 80 steps that were logarithmically distributed over the field range were measured with a Quantum Design Magnetic Properties Measurement System (MPMS XL-5, with a sensitivity of 5.0 × 10^−10^ Am^2^). A maximum field of 5 T was applied to determine the coercivity of remanence (*B*_cr_) at room temperature. The IRM acquired at 5 T is labeled as IRM_@5T_. IRM acquisition curves were decomposed into magnetic coercivity components following the method of Kruiver *et al.*^58^ in which it is assumed that the IRM acquisition curves are a linear sum of components represented by cumulative log-Gaussian functions. Subsequently, hysteresis loops for all samples were measured at room temperature using a MPMS XL-5 system with a maximum field of 5 T with 190 field steps. *B*_c_, *M*_s_, and *M*_r_ were calculated from the loops. First-order reversal curve (FORC) diagrams^59^ were acquired using a Princeton Measurements Corporation vibrating sample magnetometer (Micromag VSM 3900) at room temperature. A total of 180 FORCs were measured with saturation field up to 2 T and a field increment of 10.2 mT. FORC diagrams were processed using the FORCinel version 1.18 software^60^ with smoothing factor of 7.

## Additional Information

**How to cite this article**: Jiang, Z. *et al.* Control of Earth-like magnetic fields on the transformation of ferrihydrite to hematite and goethite. *Sci. Rep.* **6**, 30395; doi: 10.1038/srep30395 (2016).

## Figures and Tables

**Table 1:** IRM component analysis of samples grown in different applied fields.

IRM_L_				IRM_H_		
Sample name	*B*_1/2_*L*(mT)	DP	IRM Contribution (%)	*B*_1/2_*H* (mT)	DP	IRM Contribution (%)
F_0 μT_	251	0.3	18	1122	0.36	82
F_15.4 μT_	446	0.3	10	1259	0.34	90
F_29.1 μT_	398	0.3	16	1000	0.28	84
F_43.6 μT_	251	0.3	5	891	0.3	95
F_58.8 μT_	224	0.3	9	891	0.35	91
F_73.2 μT_	224	0.3	8	891	0.3	92
F_104.4 μT_	158	0.27	7	602	0.35	93
F_104.4 μT_b	316	0.27	5	631	0.35	95

*B*_1/2L_, *B*_1/2H_ are the *B*_1/2_ (mT) (the median field at which half of the IRM is reached) of coercivity components L (IRM_L_) and H (IRM_H_), respectively. DP is the dispersion parameter for each IRM component. The IRM contribution (%) is the contribution of each IRM component to the total IRM.
